# Supplementary material for: Determinants of Adverse Perinatal Outcomes in Ibadan, Nigeria: The influence of maternal lifestyle
Source: PLOS Glob Public Health. 2025 Jan 31;5(1):e0004199. doi: 10.1371/journal.pgph.0004199 (PMC11785315; doi:10.1371/journal.pgph.0004199)
Supplement: S1 Text — (DOCX) [file pgph.0004199.s001.docx]

| **Table A . Background factors (sociodemographic, obstetrical, clinical factors associated with adverse perinatal outcomes among pregnant women in Ibadan Nigeria.** | | | | | | | | | | | | |
| --- | --- | --- | --- | --- | --- | --- | --- | --- | --- | --- | --- | --- |
| **Variables** | **Low Birth Weight** | | | | **Preterm Birth** | | | | **Birth Asphyxia** | | | |
| **Sociodemographic characteristics** | **Total** | **LBW +** | **LBW-** | **P-value (X^2^)** | **Total** | **PTB +** | **PTB -** | **P-value (X^2^)** | **Total** | **BA +** | **BA -** | **P-value (X^2^)** |
| **Maternal age group** |  |  |  |  |  |  |  |  |  |  |  |  |
| < 35years | 955 | 7.855 (75) | 880 (92.15) | 0.165 (1.925) | 982 | 142(14.46) | 840 (85.54) | 0.532 (0.390) | 698 | 111 (15.90) | 587 (84.10) | 0.497 (0.4623) |
| ≥ 35years | 245 | 26 (10.61) | (219) 89.39 |  | 256 | 41 (16.02) | 215 (83.98) |  | 166 | 30(18.07  ) | 136 (81.93) |  |
| **Marital status** |  |  |  |  |  |  |  |  |  |  |  |  |
| Single | 71 | 7(9.86) | 64 (90.14) | 0.652  (0.204) | 71 | 16 (22.54) | 55 (77.46) | 0.058  (3.5943) | 53 | 8 (15.09) | 45 (84.91) | 0.803  (0.0621) |
| Married | 1,129 | 94(8.33) | 1,035 (91.67) |  | 1167 | 167 (14.31) | 1000 (85.69) |  | 811 | 133 (16.40) | 678 (83.60) |  |
| **Woman's education** |  |  |  |  |  |  |  |  |  |  |  |  |
| ≤Primary | 20 | 2(10.00) | 18(90.00) | 0.943(0.1167) | 28 | 3 (10.71) | 25 (89.29) | 0.012  (8.881) | 17 | 4 (23.53) | 13 (76.47 | 0.714  (0.6735) |
| Secondary | 278 | 24(8.63) | 254(91.37) |  | 312 | 62 (19.87) | 250 (80.13) |  | 193 | 31 (16.06) | 162 (83.94) |  |
| Tertiary | 899 | (74)8.23 | 825(91.77) |  | 895 | 117 (13.07) | 778 (86.93) |  | 651 | 105 (16.13) | 546 (83.87) |  |
| **Employment status** |  |  |  |  |  |  |  |  |  |  |  |  |
| Unemployed | 145 | 14(9.66) | 131(90.34) | 0.567  (0.3282) | 143 | 24 (16.78) | 119 (83.22) | 0.473  (0.514) | 107 | 11 (10.28) | 96 (89.72) | 0.071  (3.2615) |
| Employed | 1,055 | 87(8.25) | 968(91.75) |  | 1095 | 159 (14.52) | 936 (85.48) |  | 757 | 130 (17.17) | 627 (82.83) |  |
| **Religion** |  |  |  |  |  |  |  |  |  |  |  |  |
| Islam | 459 | 33(7.19) | 1426 (92.8) | 0.275  (1.1907) | 499 | 76 (15.23) | 423 (84.77) | 0.767  (0.088) | 327 | 46 (14.07) | 281 (5.93) | 0.165  (1.9260) |
| Christianity | 735 | (66(8.98) | 669 (91.02) |  | 732 | 107 (14.62) | 625 (85.38) |  | 532 | 94 (17.67) | 438 (82.33) |  |
| **Ethnicity** |  |  |  |  |  |  |  |  |  |  |  |  |
| Non-Yoruba | 139 | 11(7.91) | 128 (92.09) | 0.813  (0.0559) | 135 | 19 (14.07) | 116 (85.93) | 0.821  (0.051) | 103 | 19 (18.45) | 84 (81.55) | 0.545  (0.3662) |
| Yoruba | 1,058 | 90 (8.51) | 968(91.49) |  | 1101 | 163 (14.80) | 938 (85.20) |  | 758 | 122 (16.09) | 636 (83.91) |  |
| **Maternal Income** |  |  |  |  |  |  |  |  |  |  |  |  |
| <20,000 | 363 | 35(9.64) | 328 (90.36) | 0.340(2.1605) | 400 | 67 (16.75) | 83.25(333) | 0.104  (4.526) | 257 | 214 (83.27) | 43 (16.73) | 0.992  (0.0165) |
| 20,000 - 99,999 | 612 | 46 (7.52) | 566 (92.48) |  | 610 | 75 (12.30) | 87.70(535) |  | 436 | 362 (83.03) | 74 (16.97) |  |
| ≥100,000 | 74 | 4 (5.41) | 70(94.59) |  | 75 | 13 (17.33) | 82.67(62) |  | 55 | 46 (83.64) | 9 (16.36) |  |
| **Wealth tertiles** |  |  |  |  |  |  |  |  |  |  |  |  |
| Poorest | 327 | 33(10.09) | 294(89.91) | 0.440(1.6425) | 375 | 77 (20.53) | 298 (79.47) | 0.00(15.4168) | 219 | 34 (15.53) | 185 (84.47) | 0.901  (0.2092) |
| Middle | 415 | 32 (7.71) | 383 (92.29) |  | 424 | 58(13.68) | 366 (86.32) |  | 297 | 48 (16.16) | 249 (83.84) |  |
| Richest | 458 | 36 (7.86) | 422 (92.14) |  | 439 | 48 (10.93) | 391 (89.07) |  | 348 | 59 (16.95) | 289 (83.05) |  |
| **Fetal characteristics** |  |  |  |  |  |  |  |  |  |  |  |  |
| **Gender** |  |  |  |  |  |  |  |  |  |  |  |  |
| Male | 614 | 35 (5.7) | 579 (94.30) | 0.004 | 631 | 74 (11.7) | 557(88.27) | 0.036 | 4 | 75(17.08) | 364(82.92) | 0.543 |
| Female | 539 | 55 (10.2) | 484 (89.8) | 0.0902 | 559 | 89(15.92) | 470(84.08) | 4.4102 | 393 | 61(15.52) | 332(84.48) | 0.3703 |

//////////////////////////////

| **Table A. Background factors (sociodemographic, obstetrical, clinical factors associated with adverse perinatal outcomes among pregnant women in Ibadan, Nigeria. (cont.)** | | | | | | | | | | | | |
| --- | --- | --- | --- | --- | --- | --- | --- | --- | --- | --- | --- | --- |
| **Variables** | **Low Birth Weight** | | | | **Preterm Birth** | | | | **Birth Asphyxia** | | | |
| **Obstetrical characteristics** | **Total** | **LBW +** | **LBW-** | **P-value (X^2^)** | **Total** | **PTB +** | **PTB -** | **P-value (X^2^)** | **Total** | **BA +** | **BA -** | **P-value (X^2^)** |
| **Parity** |  |  |  |  |  |  |  |  |  |  |  |  |
| Nullipara | 529 | 49 (9.26) | 480 (90.74) | **0.595 (1.0385)** | 545 | 72 (13.21) | 473 (86.79) | **0.400(1.8309)** | 384 | 77 (20.05) | 307 (79.95) | **0.033(6.8127)** |
| 1-3 | 612 | 47 (7.68) | 565 (92.32) |  | 631 | 101 (16.01) | 530 (83.99) |  | 440 | 60 (13.64) | 380 (86.36) |  |
| ≥ 4 | 51 | 5 (9.80) | 46(90.20 |  | 56 | 8 (14.29) | 48 (85.71) |  | 35 | 4 (11.43) | 31 (88.57) |  |
| **Mode of delivery** |  |  |  |  |  |  |  |  |  |  |  |  |
| SVD | 715 | 47 (6.57) | 668 (93.43) | **0.011**  **(16.58)** | 755 | 97 (12.85) | 658 (87.15) | **0.013(16.1499)** | 508 | 58 (11.42) | 450 (88.58) | **0.000**  **(35.6950)** |
| Elective CS | 184 | 20 (10.87) | 164 (89.13 |  | 182 | 30 (16.48) | 152 (83.52) |  | 145 | 33 (22.76) | 112 (77.24) |  |
| Emergency CS | 215 | 30 (13.95) | 185 (86.05) |  | 217 | 44 20.28) | 173(79.72() |  | 156 | 42 (26.92) | 114 (73.08) |  |
| **History of CS** |  |  |  |  |  |  |  |  |  |  |  |  |
| No | 578 | 47 (8.13) | 531 (91.87) | **0.915 (0.0115)** | 605 | 90 (14.88) | 515 85.12) | **0.180(1.7970)** | 393 | 45 (11.45) | 348 (88.55) | **0.006**  **(7.5366)** |
| Yes | 191 | 16 (8.38) | 175 (91.62) |  | 190 | 36 (18.95) | 154 (81.05) |  | 145 | 30 (20.69) | 115 (79.31) |  |
| **History of gestational HBP** |  |  |  |  |  |  |  |  |  |  |  |  |
| No | 1023 | 92 (8.99) | 91.01(931) | **0.817(0.0534)** | 743 | 110 (14.80) | 6333 (85.20) | **0.069(3.3088)** | 509 | 69 (13.56) | 440 (86.44) | **0.322**  **(0.9821)** |
| Yes | 19 | 2 (10.53) | 17 (89.47) |  | 49 | 12 (24.49) | 37 (75.51) |  | 30 | 6 (20.00) | 24 (80.00) |  |
| **History of stillbirths** |  |  |  |  |  |  |  |  |  |  |  |  |
| Yes | 108 | 14 (12.96 | 94 (87.04) | **0.056 (3.6638)** | 112 | 26 (23.21) | 86 76.79) | **0.014(6.0757)** | 83 | 15 (18.075) | 68 (81.93) | **0.437**  **(0.6047)** |
| No | 692 | 52 (7.51) | 640 (92.49) |  | 706 | 100 (14.16) | 606 (85.84) |  | 488 | 72 (14.75) | 416 (85.25) |  |
| **History of miscarriage** |  |  |  |  |  |  |  |  |  |  |  |  |
| Yes | 288 | 27 (9.38) | 261 (90.63) | **0.325 (0.9670)** | 281 | 35 (12.46) | 246 (87.54) | **0.085(2.9723)** | 197 | 39 (19.80) | 158 (80.20) | **0.020**  **(5.410)** |
| No | 565 | 42 (7.43) | 523 (92.57) |  | 595 | 101 (16.97) | 494 (83.03) |  | 405 | 51 (12.59) | 354 (87.41) |  |
| **Chronic Medical Disease** |  |  |  |  |  |  |  |  |  |  |  |  |
| Yes | 121 | 12(9.92) | 109(90.92) | **0.531** | 127 | 17(13.39) | 110(86.61) |  | 771 | 18(19.35) | 75(80.63) | **0.402**  **0.7031** |
| No | 1079 | 89(8.25) | 990(91.75) | **0.3932** | 1111 | 166(14.94) | 945(85.06) |  | 93 | 123(15.95) | 648(84.05) |  |
| **Maternal BMI** |  |  |  |  |  |  |  |  |  |  |  |  |
| Underweight | 29 | 3(10.71) | 25(89.29) |  | 33 | 3(9.09) | 30(90.91) | 0.743  1.2423 | 18 | 3(16.67) | 15(83.33) |  |
| Normal weight | 569 | 53(9.31) | 516(90.69) |  | 599 | 93(15.53) | 506(84.47) |  | 408 | 61(14.95) | 347(85.05) | **0.321**  **3.5003** |
| Overweight | 343 | 27(78.7) | 316(92.13) |  | 346 | 49(14.16) | 297(85.84) |  | 258 | 41(15.85) | 217(84.11) |  |
| Obese | 223 | 13(5.83) | 210(94.17) |  | 222 | 32(14.41) | 190(85.59) |  | 154 | 33(21.43) | 121(78.57) |  |
| **Antenatal visits** |  |  |  |  |  |  |  |  |  |  |  |  |
| < 4 visits | 180 | 12(6.67) | 168(93.33) |  | 178 | 26(14.61) | 152(85.39) | 0.700  0.1484 | 134 | 21(15.67) | 113(84.33) | **0.904**  **0.0145** |
| ≥ 4 visits | 533 | 40(7.50) | 493(92.50) |  | 557 | 75(13.46) | 482(86.54) |  | 433 | 66(15.24) | 367(84.76) |  |

////////////////////////////////////////////////////////////////////////////

| **Table B Lifestyle and behavioural factors (physical activity, sedentary behaviors and dietary patterns) associated with adverse perinatal outcomes in Ibadan, Nigeria.** | | | | | | | | | | | | |
| --- | --- | --- | --- | --- | --- | --- | --- | --- | --- | --- | --- | --- |
| **Variables** | **Low Birth Weight** | | | | **Preterm Birth** | | | | **Birth Asphyxia** | | | |
| **Lifestyles/behaviours characteristics** | **Total** | **LBW +** | **LBW-** | **P-value (X^2^)** | **Total** | **PTB +** | **PTB -** | **P-value (X^2^)** | **Total** | **BA +** | **BA -** | **P-value (X^2^)** |
| **Alcohol use** |  |  |  |  |  |  |  |  |  |  |  |  |
| Yes | 152 | 11(7.24) | 141(92.76) | 0.575  0.3143 | 159 | 24(15.09) | 135(84.1) | 0.905  (0.0141) | 106 | 14(13.21) | 92(86.79) | 0.355  0.8568 |
| No | 1048 | 90(8.59) | 958(91.41) |  | 1079 | 159(14.74) | 920(85.26) |  | 758 | 127(16.75) | 631(83.25) |  |
| **Tobacco use** |  |  |  |  |  |  |  |  |  |  |  |  |
| Yes | 41 | 2(4.88) | 39(95.12) | 0.406  0.6896 | 45 | 8(17.78) | 37(82.22) | 0.564  (0.3327) | 31 | 7(22.58) | 24(77.42) | 0.337  0.9230 |
| No | 1159 | 99(8.59) | 1060 (91.46) |  | 1193 | 175(14.67) | 1018 (85.33) |  | 833 | 134(16.09) | 699(83.91) |  |
| **Perceived stress** |  |  |  |  |  |  |  |  |  |  |  |  |
| Low | 119 | 13(10.92) | 106(89.08) |  | 113 | 15(13.27) | 98(86.73) |  | 84 | 21(25.00) | 63(75.00) |  |
| Moderate | 891 | 64(7.89) | 827(92.82) | 0.302  2.3927 | 941 | 132(14.03) | 809(87.00) | 0.970  (0.0617) | 639 | 97(15.18) | 542(84.82) | 0.051  5.9640 |
| High | 68 | 4(5.88) | 64(94.12) |  | 67 | 9(13.43) | 58 (86.57) |  | 50 | 6(12.00) | 44(88.00) |  |
| **Depression** |  |  |  |  |  |  |  |  |  |  |  |  |
| Yes | 195 | 13(66.7) | 182(93.33) | 0.549  0.3589 | 954 | 42(20.39) | 164(79.61) | 0.006  (7.5461) | 135 | 22(16.00) | 113(83.70) | 0.792  0.0693 |
| No | 921 | 73(7.93) | 848(92.07) |  | 206 | 124(13.00) | 830(87.00) |  | 669 | 103(15.40) | 566(84.60) |  |
| **Unprescribed medicines** |  |  |  |  |  |  |  |  |  |  |  |  |
| Yes | 146 | 6(4.11) | 140(95.89) | 0.052  3.776 | 142 | 16(11.27) | 126(88.73) | 0.748  (0.1029) | 112 | 24(21.43) | 88(78.57) | 0.147  2.1069 |
| No | 347 | 32(9.22) | 315(90.78) |  | 333 | 41(12.31) | 292(87.69) |  | 256 | 39(15.23) | 217(84.77) |  |
| **Herbal** |  |  |  |  |  |  |  |  |  |  |  |  |
| Yes | 89 | 5(5.62) | 84(94.38) | 0.296  1.0932 | 93 | 17(18.28) | 76(81.72) | 0.184  (1.7661) | 75 | 17(22.67) | 58(77.33) | 0.140  2.1750 |
| No | 365 | 33(.04) | 332(90.96) |  | 349 | 45(12.89) | 304(87.11) |  | 272 | 42(15.44) | 230(84.56) |  |
| **Sedentary intensity activity** |  |  |  |  |  |  |  |  |  |  |  |  |
| Low | 398 | 29 (7.29) | 369 (92.71) | 0.148  (3.8248) | 420 | 76 (18.10) | 344 (81.90) | 0.049  (6.0514) | 276 | 44 (15.94) | 232 (84.06) | 0.756  (0.5599) |
| Medium | 383) | 41 (10.70) | 342 (89.30) |  | 410 | 50 (12.20) | 360 (87.80) |  | 268 | 41 (15.30) | 227 (84.70) |  |
| High | 419) | 31 (7.40) | 388 (92.60) |  | 408 | 57 (13.97) | 351 (86.03) |  | 320 | 56 (17.50) | 264 (82.50) |  |
| **Light intensity activity** |  |  |  |  |  |  |  |  |  |  |  |  |
| Low | 411) | 37 (9.00) | 374 (91.00) | 0.683  (0.7635) | (418) | 58 (13.88) | 360(86.12) | 0.409  (1.7859) | 295 | 58 (19.66) | 237 (80.34) | 0.130  (4.0790) |
| Medium | 391) | 29 (7.42) | 362 (92.58) |  | (407) | 68 (16.71) | 339 (83.29) |  | 280 | 38 (13.57) | 242 (86.43) |  |
| High | 398) | 35 (8.79) | 363 (91.21) |  | (413) | 57 (13.80) | 356 (86.20) |  | 289 | 45 (15.57) | 244 (84.43) |  |
| **Moderate intensity** |  |  |  |  |  |  |  |  |  |  |  |  |
| Low | 420 | 34 (8.10) | 386 (91.90) | 0.526  (1.2840) | 431 | 55 (12.76) | 376( 87.24) | 0.130  (4.0831) | 307 | 55 (17.92) | 252 (82.08) | 0.582  (1.0825) |
| Medium | 393 | 38 (938) | 355 (90.33) |  | 416 | 73 (17.55) | 343 (82.45) |  | 279 | 45 (16.13) | 234 (83.87) |  |
| High | 387 | 29 (7.49) | 358 (92.51) |  | 391) | 55 (14.07) | 336 (85.93) |  | 278 | 41 (14.75) | 237 (85.25) |  |
| **Vigorous activity** |  |  |  |  |  |  |  |  |  |  |  |  |
| Low | 741 | 63 (8.50) | 678 (91.50) | 0.950  (0.1034) | 774 | 115 (14.86) | 659 (85.14) |  | 538 | 81 (15.06) | 457 (84.94) |  |
| Medium | 183 | 16 (8.74) | 167 (91.26) |  | 186) | 28 (15.05) | 158 (84.95) |  | 133) | 23 (17.29) | 110 (82.71) |  |
| High | 276) | 22 (7.97) | 254 (92.03) |  | 278) |  | |  |  | | |  |

//////////////////////////////////////////////////////////////////////////

|  | **Table B. Lifestyle and behavioural factors (physical activity, sedentary behaviours and dietary patterns) associated with adverse perinatal outcomes in Ibadan, Nigeria. (cont.)** | | | | | | | | | | | | | | | |
| --- | --- | --- | --- | --- | --- | --- | --- | --- | --- | --- | --- | --- | --- | --- | --- | --- |
| **Variables** | | **Low Birth Weight** | | | | **Preterm Birth** | | | |  | **Birth Asphyxia** | | | | | |
|  | | **Total** | **LBW +** | **LBW-** | **P-value**  **(X^2^)** | **Total** | **PTB +** | **PTB -** | **P-value**  **(X^2^)** | **Total** | | **BA +** |  | **BA -** | **P-value (X^2^)** |  |
| **Occupation-related activity** | |  |  |  |  |  |  |  |  |  | |  |  |  |  |  |
| Low | | 425 | 37(8.71) | 388 (91.29) | 0.644  (0.8805) | 427 | 70(16.39) | 357(83.61) | 0.489  (1.4297) | 304 | | 50(16.45) |  | 254(83.55) | 0.60  (5.6257) |  |
| Medium | | 406 | 37(9.11) | 369(90.89) |  | 420 | 60(14.29) | 360(85.71) |  | 271 | | 57(19.86) |  | 230(80.14) |  |  |
| High | | 369 | 27 (7.32) | 342(92.68) |  | 391 | 53(13.55) | 338(86.45) |  | 273 | | 34(12.45) |  | 239(87.55) |  |  |
| **Transport-related activity** | |  |  |  |  |  |  |  |  |  | |  |  |  |  |  |
| Low | | 459 | 38(8.28) | 42(91.72) | 0.918  (0.1714) | 478 | 59(12.34) | 374()87.66 | 0.159  (3.6764) | 324 | | 58(17.90) |  | 266(82.10) | 0.060  (5.6257) |  |
| Medium | | 359 | 32(8.91) | 327(91.09) |  | 380 | 62(16.32) | 340(83.68) |  | 271 | | 42(15.50) |  | 229(84.50) |  |  |
| High | | 382 | 31(8.12) | 351(91.88) |  | 380 | 62(16.32) | 341(83.68) |  | 269 | | 41(15.24) |  | 228(84.76) |  |  |
| **Household/caregiving activity** | |  |  |  |  |  |  |  |  |  | |  |  |  |  |  |
| Low | | 405 | 32(7.90) | 373(92.10) | 0.633  (0.9143) | 424 | 50(11.79) | 395(88.21) | 0.099  (4.6286) | 288 | | 50(17.36) |  | 238(82.64) | 0.835  (0.3618) |  |
| Medium | | 359 | 31(7.85) | 364(92.15) |  | 405 | 65(16.05) | 338(83.95) |  | 276 | | 43(15.58) |  | 233(84.42) |  |  |
| High | | 400 | 38(9.50) | 362(90.50) |  | 409 | 68(16.63) | 322(83.37) |  | 300 | | 48(16.00) |  | 252(84.00) |  |  |
| **Sports activity** | |  |  |  |  |  |  |  |  |  | |  |  |  |  |  |
| Low | | 432 | 33(7.64) | 399(92.36) | 0.503  (1.3725) | 455 | 60(13.19) | 395(86.81) | 0.288  (2.4873) | 313 | | 46(14.70) |  | 267(85.30) | 0.617  (0.9649) |  |
| Medium | | 390 | 31(7.95) | 359(92.05) |  | 395 | 57(14.43) | 338(85.57) |  | 276 | | 47(17.03) |  | 229(82.97) |  |  |
| High | | 378 | 37(9.79) | 341(90,21) |  | 388 | 66(17.01) | 322(82.99) |  | 275 | | 48(17.45) |  | 227(82.55) |  |  |
| **Dietary patterns** | |  |  |  |  |  |  |  |  |  | |  |  |  |  |  |
| **Protein-rich diet and non-alcoholic beverages** | |  |  |  |  |  |  |  |  |  | |  |  |  |  |  |
| Low | | 411 | 33(8.03) | 378(91.97) | 0.072  (5.2533) | 416 | 74(17.79) | 342(82.21) | 0.005  (10.5505) | 299 | | 50(16.72) |  | 249(83.28) | 0.877  (0.2616) |  |
| Medium | | 408 | 44 (10.73) | 364(89.22) |  | 426 | 69(16.20) | 357(83.80) |  | 298 | | 46(15.44) |  | 252(84.56) |  |  |
| High | | 381 | 24(6.S0) | 357(93.70) |  | 396 | 40(10.10) | 356(89.90) |  | 267 | | 45(16.85) |  | 222(83.15) |  |  |
| **Fruits Diet** | |  |  |  |  |  |  |  |  |  | |  |  |  |  |  |
| Low | | 413 | 31(7.51) | 382(92.49) | 0.692  (0.7376) | 422 | 60(14.22 | 362(85.78) | 0.009  (9.4471) | 306 | | 43(14.05) |  | 263(85.95) | 0.364  (2.0198) |  |
| Medium | | 393 | 34(8.65) | 359(91.35) |  | 415 | 78(18.80) | 337(81.20) |  | 284 | | 52(18.31) |  | 232(81.69) |  |  |
| High | | 394 | 36(9.14) | 358(90.86) |  | 401 | 45(11.22) | 356(88.78) |  | 274 | | 46(83.21) |  | 228(83.21) |  |  |
| **Typical diet with alcohol** | |  |  |  |  |  |  |  |  |  | |  |  |  |  |  |
| Low | | 408 | 33(8.09) | 375(91.91) | 0.955  (0.0929) | 430 | 58(13.49) | 372(86.51) | 0.156  (3.7145) | 310 | | 48(15.48) |  | 262(84.52) | 0.795  (0.4579) |  |
| Medium | | 404 | 35(8.66) | 369(91.34) |  | 404 | 71(17.57) | 333(82.43) |  | 280 | | 49(17.50) |  | 231(82.50) |  |  |
| High | | 388 | 33(8.51) | 355(91.49) |  | 404 | 54(13.37) | 350(86.63) |  | 274 | | 44(16.06) |  | 230(83.94) |  |  |
| **Legumes** | |  |  |  |  |  |  |  |  |  | |  |  |  |  |  |
| Low | | 447 | 42(9.40) | 405(90.60) | 0.350  (2.0970) | 442 | 60(13.57) | 382(86.43) | 0.256  (2.7264) | 335 | | 59(17.61) |  | 276(82.39) | 0.209  (3.1320) |  |
| Medium | | 398 | 27(6.78) | 371(93.22) |  | 421 | 72(17.10) | 349(82.90) |  | 288 | | 38(13.19) |  | 250(86.81) |  |  |
| High | | 355 | 32(9.01) | 323(90.99) |  | 375 | 51(13.60) | 324(86.40) |  | 241 | | 44(18.26) |  | 197(81.74) |  |  |
| **Refined grains** | |  |  |  |  |  |  |  |  |  | |  |  |  |  |  |
| Low | | 397 | 31(7.81) | 366(92.19) | 0.758  (0.5545) | 412 | 58(14.08) | 354(85.92) | 0.143  (3.8932) | 284 | | 48(16.90) |  | 236(83.10) | 0.933  (0.1389) |  |
| Medium | | 401 | 37(9.23) | 364(90.77) |  | 418 | 73(17.46) | 345(82.54) |  | 292 | | 46(15.75) |  | 246(84.25) |  |  |
| High | | 402 | 33(8.21) | 369(91.79) |  | 408 | 52(12.75) | 356(87.25) |  | 288 | | 47(16.32) |  | 241(83.68) |  |  |
