# Supplementary material for: Determinants of Adverse Perinatal Outcomes in Ibadan, Nigeria: The influence of maternal lifestyle
Source: PLOS Glob Public Health. 2025 Jan 31;5(1):e0004199. doi: 10.1371/journal.pgph.0004199 (PMC11785315; doi:10.1371/journal.pgph.0004199)
Supplement: Attachment — Submitted filename: Review - Manuscript Number PGPH-D-24-00167.docx [file pgph.0004199.s002.docx]

Review - Manuscript Number PGPH-D-24-00167

"DETERMINANTS OF ADVERSE PERINATAL OUTCOMES IN IBADAN, NIGERIA: THE INFLUENCE OF MATERNAL LIFESTYLE"

This paper refers to a cohort of pregnant women with the analysis of lifestyle status and adverse perinatal outcomes in Nigeria. I appreciate the editorial board's invitation to review this paper and congratulate the authors for the study. Please find some comments below:

The **abstract** does not mention the results regarding the lifestyle determinants. Since this is the leading study objective, these findings should be shown in this section.

**Introduction**

In line 75, there appears to be an asterisk that could be a typographical error. This needs to be addressed for the sake of clarity.

Line 92 – The mortality rate is not adequately expressed. The definition for this indicator is the estimated total number of deaths in a population of a given sex and age, divided by the total number of this population, expressed per 100,000 or 1,000 population, for a given year in a given country, territory, or geographic area.

Line 102 – ANC was not previously named. This should be done before using the abbreviation.

**Results**

Line 258 – Although minimum, the mean age shown here differs from the abstract (29.96 ± 5.2 years).

Tables 1 and 2 must present the univariate statistical analysis, in addition to the logistic regression shown in Tables 3 and 4, to better understand the associations between the variables.

The text description of the results should be improved to clarity. Ideally, the text must highlight the main results and guide readers to the tables where they can find the information and statistics. The text is now a bit messy and impairs the flow of reading and comprehension.

**Across all sections**

Attention should be given to Capital Letters in some words without grammatical justification.

The **discussion** section has better text organization and grammar structure than others, facilitating comprehension. Other previous sections should be reviewed to follow the same structure.

The **acknowledgment** is written in the first person, which may not be adequate as there are other authors in this study. Also, the **Contribution of Authors** section is empty.
