# Supplementary material for: Determinants of Adverse Perinatal Outcomes in Ibadan, Nigeria: The influence of maternal lifestyle
Source: PLOS Glob Public Health. 2025 Jan 31;5(1):e0004199. doi: 10.1371/journal.pgph.0004199 (PMC11785315; doi:10.1371/journal.pgph.0004199)
Supplement: Attachment — Submitted filename: Point by point response to the Reviewers comments_PGPH ADR.docx [file pgph.0004199.s003.docx]

Review - Manuscript Number PGPH-D-24-00167

"DETERMINANTS OF ADVERSE PERINATAL OUTCOMES IN IBADAN, NIGERIA: THE INFLUENCE OF MATERNAL LIFESTYLE"

This paper refers to a cohort of pregnant women with the analysis of lifestyle status and adverse perinatal outcomes in Nigeria.

1. The **abstract** does not mention the results regarding the lifestyle determinants. Since this is the leading study objective, these findings should be shown in this section.

- Lifestyle is now mentioned in the abstract.
- *“Of the lifestyle factors examined, only a high protein diet with a non-alcoholic beverage dietary pattern had a statistically significant association with preterm birth [AOR: 0.50, 95%CI (1.08 – 3.52)]. However, lifestyle factors had no significant association with LBW and birth asphyxia in our study. Understanding these risk factors can help policymakers and healthcare professionals create cost-effective interventions to curtail the burden of APO in Nigeria. Further research is needed to explore lifestyle factors and APO within the Nigerian context”.*

**Introduction**

2. In line 75, there appears to be an asterisk that could be a typographical error. This needs to be addressed for the sake of clarity.

- The asterisk is replaced with a reference.

3. Line 92 – The mortality rate is not adequately expressed. The definition for this indicator is the estimated total number of deaths in a population of a given sex and age, divided by the total number of this population, expressed per 100,000 or 1,000 population, for a given year in a given country, territory, or geographic area.

- Corrected
- *While 1 in every 300 neonates dies in HIC, 1 in 36 die in sub-Saharan Africa (sSA) [10]. Moreover, West Africa reports the highest perinatal mortality rate of 65.1 per 1000 total births compared to 49.9 per 1000 total births and 56 per 1000 total births in East Africa and South Africa, respectively [9].*

4. Line 102 – ANC was not previously named. This should be done before using the abbreviation.

- Antenatal care (ANC) first mentioned

**Results**

5. Line 258 – Although minimum, the mean age shown here differs from the abstract (29.96 ± 5.2 years).

- Corrected (30.0 ± 5.2 years)

.

6. Tables 1 and 2 must present the univariate statistical analysis and the logistic regression shown in Tables 3 and 4 to understand the associations between the variables better.

The text description of the results should be improved to clarity. Ideally, the text must highlight the main results and guide readers to the tables where they can find the information and statistics. The text is now a bit messy and impairs the flow of reading and comprehension.

- Corrected and recast

**Across all sections**

7. Attention should be given to Capital Letters in some words without grammatical justification.

- All unnecessary Capital Letters have been removed.

8. The **discussion** section has better text organisation and grammar structure than others, facilitating comprehension. Other previous sections should be reviewed to follow the same structure.

- All the other sections have been revised, including the introduction, methods, and results.

9. The **acknowledgement** is written in the first person, which may not be adequate as there are other authors in this study. Also, the **Contribution of Authors** section is empty.

- Acknowledgement is rewritten in the third person – we
- *We appreciate our research team – research nurses, laboratory scientists, numerous research assistants, and data personnel- for their dedication, support, and hard work. We also appreciate the health workers –doctors, nurses, clinic and record staff of the various health facilities for their cooperation and support in the four facilities: University College Hospital, Adeoyo Maternity Teaching Hospital, Jericho Specialist Hospital, and Saint Mary Catholic Hospital Oluyoro, Ibadan. We appreciate the input of CARTA (Consortium for Advanced Research Training for Africa) in all its training, care, support, oversight, funding, and sponsorship efforts.*

***Contribution of Authors – INPUTED INTO THE PGPH PLATFORM***

*IAA designed and conducted the study and analysed the data. COU wrote the initial draft of the manuscript. IAA, COU, KA and BMG interpreted the data. IAA, COU, KA and BMG reviewed and critically revised the manuscript. IAA and COU finalised the manuscript. All authors read and approved the final manuscript.*
