# Supplementary material for: Determinants of Adverse Perinatal Outcomes in Ibadan, Nigeria: The influence of maternal lifestyle
Source: PLOS Glob Public Health. 2025 Jan 31;5(1):e0004199. doi: 10.1371/journal.pgph.0004199 (PMC11785315; doi:10.1371/journal.pgph.0004199)
Supplement: Attachment — Submitted filename: Point by Point response to Reviewer Comments_APO.docx [file pgph.0004199.s004.docx]

**POINT BY POINT RESPONSE TO REVIEWER COMMENTS:

REVIEWER 1**

In their study, Adeoye et al. reported the determinants of adverse pregnancy outcomes (APOs) in Nigeria and their association with maternal lifestyle. The data presented are important and timely, as APOs significantly impact perinatal and neonatal survival, increasing the risk of child mortality, developmental disabilities, and lifelong ill health. Countries in sub-Saharan Africa bear a disproportionate share of the global burden of APOs, yet few studies from the region have reported on the factors associated with these outcomes. However, the authors should consider addressing the following issues to further enhance the value of the paper.

1. The abstract is lengthy; please consider condensing it to enhance readability.

- DONE
- Abstract now shortened

2. In the methods section, the analysis plan needs to be more explicit in explaining how the multivariate logistic regression model was built to calculate the adjusted odds ratios.

- DONE
- ***Stated as follows***
- *Statistical analysis was performed using Stata version 13. Descriptive statistics summarised continuous data with mean and standard deviation and frequency and percentages for categorical data. The prevalence of APO and the specific causes – LBW, PTB and birth asphyxia were displayed graphically with bar graphs. The associations between respondent's background (Sociodemographic, maternal, obstetrical and medical) and lifestyle factors (like dietary patterns, physical activity, alcohol and smoking) and Adverse Perinatal Outcomes were tested using the chi-square test. Variables that were statistically significant at 5% level (history of stillbirth, mode of delivery, sex of the newborn, occupationally related physical activity) were subjected to multiple logistic regression analyses. Also the associations between respondent's background and lifestyle factors and the specific causes of APO - LBW, PTB and birth asphyxia examined using chi-square test (in supplementary material), binary and multiple logistic regression analyses. Variables that were statistically significant at 5% level were subjected to multiple logistic regression analyses as follows LBW (sex of the newborn, mode of delivery, a history of gestational hypertension); PTB (maternal income, wealth index. Mode of delivery, history of still birth, antepartum depression, sedentary activity, moderate intensity activity, household activity, protein rich diet and non- alcoholic beverages); birth asphyxia (parity, mode of delivery, history of miscarriage). Unadjusted and adjusted odds ratios and 95% confidence intervals (CI) and p-values were reported.*

3. Table 1 and elsewhere indicate that percentages were calculated using a denominator of 1,339, which does not seem appropriate, as Figure 1 shows that newborn outcomes could not be tracked for 60 deliveries. Please correct the denominators or clarify why this denominator is being used.

- DONE
- The earlier flowchart was a mistake, is not for the current study. Data is available for 1339 women who had hospital delivery
- New flowchart provided

4. Please include appropriate statistical test values in Tables 1 and 2 to indicate whether there are any significant differences in the prevalence of adverse pregnancy outcomes (APOs) between the sub-groups of mothers or neonates.

- DONE
- The appropriate statistical test values (chi square tests and p-values) now placed in Tables 1 and 2

5. In Tables 1 and 2, please indicate in the footnotes if more than 5% of data are missing for any particular variable.

- DONE
- The footnotes now indicates where more than 5% of data are missing

6. The paper would flow better if the findings related to lifestyle determinants are presented first, as this is the primary objective of the manuscript.

- DONE
- lifestyle determinants of APO now presented first.

7. In Tables 3 and 4, please add the number of events for each category, as knowing the number of APOs for each category is essential to understanding the significance of the data.

- DONE
- Tables 4a and 5a that describes the number and proportions in each category test of associations now presented as a supplementary material

8. While the data on each adverse outcome in Tables 3 and 4 are valuable, it would have greater policy impact if the effect of each determinant were calculated to assess its overall impact on APOs.

- DONE
- Additional analysis on Determinants of APO as a construct and presented on a new Table 3. Former Tables 3 and 4 now Tables 4 and 5

9. In line 526, it states that physical activities increase the odds of preterm birth (PTB), but the data do not support this finding. This could mislead the reader, as some previous reports suggest that moderate physical work may reduce adverse pregnancy outcomes (APOs).

- DONE -
- The association between physical activities and preterm birth (PTB) not significant AND now discussed as such. - RECAST
- *In this study, physical activity had no significantly association with PTB on in the adjusted model.Notably, scientists have reported conflicting results on the association between physical activity and PTB; some have reported no association [56, 57], and a few have reported a positive association [58] and inverse association [57].*

10. In lines 368-372, presenting only p-values is insufficient, as the determinants can affect the outcome in either direction. Please include the associated odds ratios (OR) for each variable.

- CORRECTED
- The associated unadjusted odds ratios (UOR), 95% confidence intervals and p-values now reported
- *The prevalence of PTB was 11.8% in this study. On univariate analysis, the significant determinants were income ( UOR=0.6995% CI: (0.48-0.99) p=0.043), wealth index (UOR=0.48, 95% CI: (0.32-0.71) p<0.001), history of stillbirth (UOR=1.84, 95% CI: (1.13-2.99) p=0.014), emergency caesarean section (UOR=1.63, 95% CI: (1.12-2.38) p=0.011), spontaneous vagina delivery UOR= 0.70, 95% CI: (0.51-0.96) p= 0.027) , antepartum depression ( UOR=1.73, 95% CI: (1.17-2.55) p=0.006), being born female ( UOR=1.43, 95% CI: (1.03-2.00) p=0.034), and a high protein diet (UOR=0.52, 95% CI: (0.34-0.78) p=0.002). Physical activity had a significant association with PTB in the unadjusted model: medium level of moderate-intensity physical activity [UOR=1.5;95% CI: (1.01-2.17); p-value=0.043], high levels of household/caregiving activity; [UOR=1.5; 95% CI:(1.03-2.27); p-value=0.035] had positive associations with PTB while medium level of sedentary-intensity physical activity [UOR=0.6; 95% CI:(0.43-0.93); p-value<0.021] was inverse. The associations became insignificant after adjustment*.

11. Please highlight some limitations of the paper that may affect the outcomes of the findings.

- DONE

12. In the conclusion, the authors emphasized promoting a healthy lifestyle without specifying which aspects of lifestyle should be promoted. Please clarify those.

- DONE
